# Trispecific antibody targeting HIV-1 and T cells activates and eliminates latently-infected cells in HIV/SHIV infections

**DOI:** 10.1038/s41467-023-39265-z

**Published:** 2023-06-22

**Authors:** Wanwisa Promsote, Ling Xu, Jason Hataye, Giulia Fabozzi, Kylie March, Cassandra G. Almasri, Megan E. DeMouth, Sarah E. Lovelace, Chloe Adrienna Talana, Nicole A. Doria-Rose, Krisha McKee, Sabrina Helmold Hait, Joseph P. Casazza, David Ambrozak, Jochen Beninga, Ercole Rao, Norbert Furtmann, Joerg Birkenfeld, Elizabeth McCarthy, John-Paul Todd, Constantinos Petrovas, Mark Connors, Andrew T. Hebert, Jeremy Beck, Junqing Shen, Bailin Zhang, Mikhail Levit, Ronnie R. Wei, Zhi-yong Yang, Amarendra Pegu, John R. Mascola, Gary J. Nabel, Richard A. Koup

**Affiliations:** 1 Vaccine Research Center, National Institute of Allergy and Infectious Diseases, National Institutes of Health, Bethesda, MD USA; 2 Sanofi, 640 Memorial Dr., Cambridge, MA 02139 USA; 3 ModeX Therapeutics Inc., 22 Strathmore Road, Natick, MA 01760 USA; 4 NIAID, NIH, Bethesda, MD 20892 USA; 5 Present Address: Perspix Biotech GmbH, FiZ Frankfurt Innovation Center Biotechnology, Altenhoeferallee 3, 60438 Frankfurt, Germany; 6 Present Address: Department of Laboratory Medicine and Pathology, Institute of Pathology, Lausanne University Hospital (chuv) and University of Lausanne, Lausanne, Switzerland; 7 Present Address: ModeX Therapeutics Inc., 22 Strathmore Road, Natick, MA 01760 USA

**Keywords:** Medical research, Diseases, Viral reservoirs, Antiviral agents, Retrovirus

## Abstract

Agents that can simultaneously activate latent HIV, increase immune activation and enhance the killing of latently-infected cells represent promising approaches for HIV cure. Here, we develop and evaluate a trispecific antibody (Ab), N6/αCD3-αCD28, that targets three independent proteins: (1) the HIV envelope via the broadly reactive CD4-binding site Ab, N6; (2) the T cell antigen CD3; and (3) the co-stimulatory molecule CD28. We find that the trispecific significantly increases antigen-specific T-cell activation and cytokine release in both CD4^+^ and CD8^+^ T cells. Co-culturing CD4^+^ with autologous CD8^+^ T cells from ART-suppressed HIV^+^ donors with N6/αCD3-αCD28, results in activation of latently-infected cells and their elimination by activated CD8^+^ T cells. This trispecific antibody mediates CD4^+^ and CD8^+^ T-cell activation in non-human primates and is well tolerated in vivo. This HIV-directed antibody therefore merits further development as a potential intervention for the eradication of latent HIV infection.

## Abstract (web summary)

One of the main hurdles to curing HIV infection are viral reservoirs. Here, the authors develop a trispecific antibody and demonstrate its ability to simultaneously activate and target latently HIV−1 infected cells for elimination by T cells as an alternative strategy for HIV cure.

## Introduction

Combination antiretroviral therapy (cART) against HIV/AIDS has proven to be extremely effective in reducing HIV viremia, stopping disease transmission, and delaying the progression of the disease^1–6^. However, curing HIV infection remains elusive even with such effective anti-viral treatment. The underlying problem is related to the life cycle of HIV where there is a latent stage in which integrated replication-competent provirus remains dormant and cannot be eradicated by cART treatment^7–9^, yet can be re-activated upon stimulation^10–16^. Elimination of HIV-infected cells in vivo may require a three-step process. First, the latent HIV provirus must be activated in T cells^17–19^. The second step is to eliminate those activated cells that produce HIV (through targeting by cytotoxic T cells) and the third step is to block new infections with cART^17–20^. Current cure efforts employ a “shock and kill” strategy, which aims to purge the HIV latent reservoirs using latency-reversing agents (LRAs) to induce viral reactivation that can lead to immune cell recognition and clearance of latently-infected cells^19,21–25^, but they do not directly stimulate killing of the latently-infected cells.

We previously described a bispecific antibody that combined recognition of HIV Env with T cell activation via anti-CD3, as a means to promote CD8^+^ T-cell-mediated lysis of both constitutively and latently-infected cells^26^. However, optimal T-cell activation and effector function requires both a primary signal through the interaction of peptide-loaded MHC on antigen-presenting cell (APC) and the T-cell receptor (TCR) complex on T cells, and a co-stimulatory signal provided by the interaction of CD80/CD86 on APC and CD28 on T cells. Without this second signal, agonistic CD3 signaling can lead to activation induced cell death^27–30^. Prior studies have also shown that mitogenic anti-CD3 can mimic the MHC/TCR interaction, and agonistic anti-CD28 can promote signaling and improved survival via the CD80/CD86/CD28 pathway^31^.

Substantial evidence in human studies suggest that an anti-CD3 antibody when used along with an anti-CD28 antibody leads to activation of cytotoxic T cells and of the latent reservoir of HIV-infected CD4^+^ T cells^10,11,32^. Similarly, promising preclinical data from the use of a recently developed trispecific antibody, αCD38/αCD3-αCD28, for myeloma therapy confirm that the combination of anti-CD3 and anti-CD28 can enhance both T cell activation and tumor targeting^33^.

In this study, we investigated whether the trispecific antibody platform could be used to design improved therapies for the eradication of HIV. Specifically, we explored whether a single molecule that targets three independent targets: (1) the CD4 binding site (CD4bs) of HIV envelope via N6 for targeting HIV^+^ cells, (2) an anti-CD3 for T cell activation/recruitment, and (3) an anti-CD28 to providing co-stimulatory signal for better T cell activation/survival, could reactivate virus and promote lysis of latently-infected cells.

## Results

### Production and characterization of trispecific N6/αCD3-αCD28

**Fig. 1:**
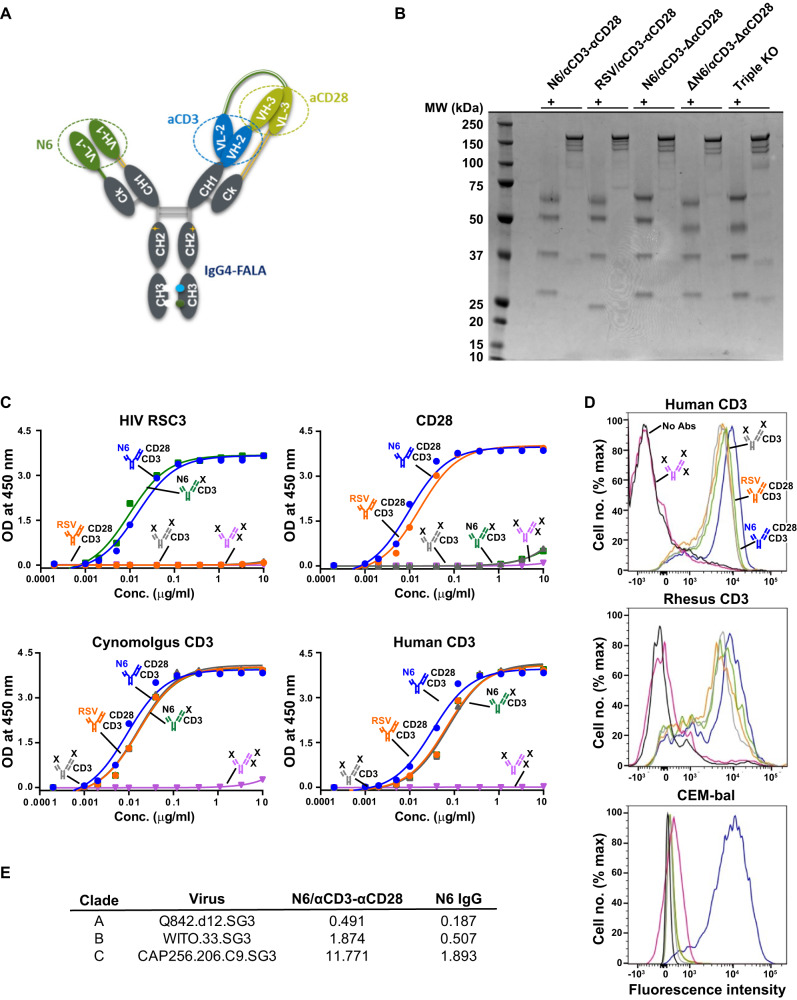
Construction and characterization of trispecific antibodies. **A** Configuration of the trispecific antibody. Colored shades (blue or green) denote variable heavy and light chain domains, whereas grey shades denote constant heavy and light chain domains. VH/VL, variable fragments. **B** The indicated trispecific antibodies were analysed by SDS-PAGE once with gel image showing the bands at the appropriate molecular sizes for the two heavy chains above 50 kDa and two light chains above 25 kDa under reducing conditions (+) and a single predominant 175 kDa band under non-reducing conditions (−). **C** ELISAs showing that N6/αCD3-αCD28 binds to the CD4-binding site (CD4bs) of HIV Env, CD28 and CD3. The N6/αCD3-αCD28 and control trispecific antibodies at increasing five-fold concentrations were allowed to bind to either a resurfaced HIV Env fragment containing the CD4bs, CD28, human or cynomolgus CD3 that was coated on ELISA plates and the bound antibodies were detected using a HRP-conjugated anti-IgG probe. The data represents the mean values from two biologically independent experiments. **D** N6/αCD3-αCD28 binds to T cells and HIV Env on the cell surface. Human T cells, rhesus T cells and HIV-infected CEM cells were incubated with trispecific N6/αCD28-αCD3 and the control antibody, and bound antibodies were detected by a fluorescein isothiocyanate-conjugated anti-IgG probe. and (**E**) Neutralization IC50 titres (µg ml^−1^) for the indicated trispecific N6/αCD3-αCD28 and control N6 protein against three representative HIV strains from clades (**A**, **B** and **C**). Source Data are provided as a Source Data file.

We generated a novel trispecific N6/αCD3-αCD28 construct by integrating a CD4bs bnAb N6, an anti-CD3 and an anti-CD28 in CODV format as previously described to be able to activate T cells in the absence or presence of tumor antigen CD38 (Fig. 1A)^33^. Related control constructs that had one, or two, specificities knocked out (ΔN6/αCD3-ΔαCD28, N6/αCD3-ΔαCD28) were developed as controls. A third control (RSV/αCD3-αCD28) was also developed by replacing N6 with a specificity directed to an irrelevant protein, the respiratory syncytial virus (RSV) F protein. Trispecific N6/αCD28-αCD3 was produced in Expi 293 cells, and purified using Protein A affinity column, followed by size exclusion chromatography and showed the expected monomeric molecular weights and composition when run on SDS-PAGE gels under both reducing and non-reducing conditions (Fig. 1B). N6/αCD28-αCD3 demonstrated the ability to bind to a resurfaced stabilized HIV core protein, RSC3^34^, CD3, and CD28 proteins (Fig. 1C), as expected. It also showed the neutralizing activity against HIV pseudoviruses (Fig. 1D). Further characterization for cell surface binding of N6/αCD3-αCD28 showed the predicted pattern of binding to CD3 on human/rhesus T cells and HIV Env on HIV-infected CEM-NKr-CCR5 cells using flow cytometry (Fig. 1E). The relevant negative control constructs also displayed the expected non-reactivity with HIV Env, CD3 and CD28 (Fig. 1C, D).

### In vitro activation and lysis of HIV latently-infected cells

**Fig. 2:**
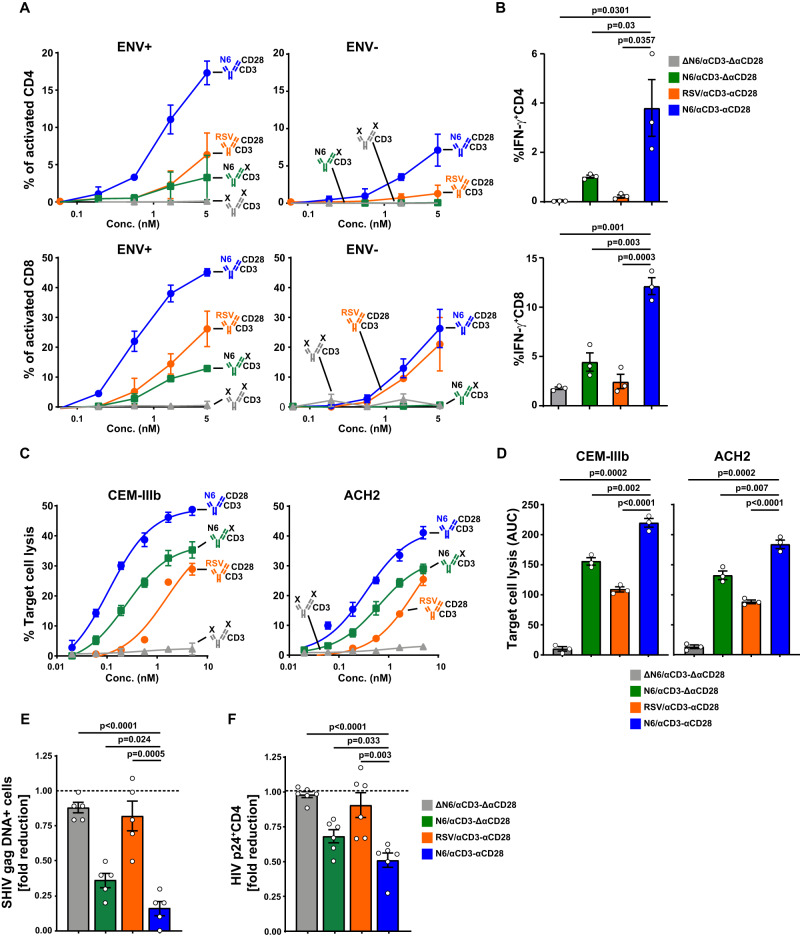
N6/αCD3-αCD28 enhances the T cell activation and T cell mediated lysis of SHIV- and HIV- infected cells. **A**, **B** HIV dependent activation of T cells by N6/αCD3-αCD28. Effector PBMC cells were co-cultured with target cells that were either uninfected or HIV-infected CEM cells (indicated by ENV- and ENV+) in the presence of the trispecific antibodies and Brefeldin A for 16 h. The percentage of activated CD4^+^ and CD8^+^ T cells expressing both CD69 and CD25 (**A**) and intracellular IFN-γ (**B**) were measured using flow cytometry. The data were plotted as the mean ± SEM (*n* = 3 biological replicates). **C**, **D** Targeted lysis of HIV infected cell lines CEM-IIIb and ACH2 by N6/αCD3-αCD28. The HIV infected cells were co-cultured with SHIV^+^ LN CD8^+^ T cells which served as effector cells, in the presence of trispecific antibodies for 12 h. Percent lysis of the infected cells was measured using flow cytometry after staining with live/dead and apoptotic cell markers. Representative dose response data (**C**) and area under the curve (AUC) analysis (**D**) from three independent experiments. **E** Reduction in the levels of SHIV *gag* DNA in CD4^+^ T cells from SHIV-infected LN by N6/αCD3-αCD28. Sorted CD4^+^ and CD8^+^ T cells that served as target and effectors, respectively, were co-cultured with trispecific antibodies for 12 h. CD4^+^ T cells were then resorted from the co-culture for quantification of SHIV g*ag* DNA. The data were plotted as the mean ± SEM (*n* = 5 biological replicates). **F** Reduction in the number of latently-infected CD4^+^ T cells by N6/αCD3-αCD28. Resting CD4^+^ T cells (CD4^+^CD25^−^CD69^−^) were sorted from PBMCs and infected with HIV BaL after culture with CCL19 for 3 days. These CD4^+^ T cells were then co-cultured with allogeneic CD8^+^ T cells and trispecific antibodies for 14 h. Intracellular p24 expression of CD4^+^ T cells was then measured by flow cytometry. The data were plotted as the mean ± SEM (*n* = 6 biological replicates). Statistical significance was measured by unpaired, two-tailed Student’s *t*-test with *P* values less than 0.05 considered significant (**B**, **D**, **E**, **F**). Source Data are provided as a Source Data file.

To investigate whether N6/αCD3-αCD28 could stimulate T cell activation with or without exposure to HIV, we analyzed the response of human CD4^+^ and CD8^+^ T cells in the presence of target cells that were either uninfected or HIV-infected CEM cells (indicated by ENV− and ENV+) with the active trispecific and relevant negative control constructs. In HIV-infected CEM cells, N6/αCD3-αCD28 stimulated a significant, dose-dependent activation of CD4^+^ T cells, as evidenced by increased CD25 and CD69 expression. The level of activation in the presence of uninfected CEM cells was much less at the same concentration (Fig. 2A). In the presence of HIV-infected CEM cells, the control construct RSV/αCD3-αCD28 showed similar level of activation as N6/αCD3-αCD28 in the presence of uninfected CEM cells (Fig. 2A), again suggesting that αCD3-αCD28 could activate CD4^+^ T cells in absence of N6 binding to HIV Env. Examination of the CD8 response in the presence of HIV-infected CEM cells revealed a similar pattern of stimulation with a higher percentage of reactive cells when stimulated with N6/αCD3-αCD28 as compared to the control constructs (Fig. 2A). In the presence of uninfected CEM cells, higher doses of the trispecific Ab were required to achieve activation (Fig. 2A). These analyses document that stimulation by N6/αCD3-αCD28 is antigen-specific and dose-dependent. Although some nonspecific stimulation can be observed, higher doses were needed for the antigen-independent immune stimulation in vitro.

Incubation of CD4^+^ T cells in the presence of HIV-infected CEM cells with N6/αCD3-αCD28 stimulated a 10-fold increase in interferon (IFN)-γ intracellular cytokine staining (Fig. 2B) compared with 3.2-fold in the presence of uninfected CEM cells relative to unstimulated CD4^+^ T cells (Supplementary Fig. 1). In contrast, the control constructs showed minimal effects (Fig. 2B). Examination of the CD8 response revealed a similar trend in IFN-γ intracellular cytokine staining, with a significantly higher percentage of reactive cells in this population when N6/αCD3-αCD28 was used (Fig. 2B; *P* = 0.0003–0.003, Student’s *t*-test). The other negative control constructs also produced minimal activation. In presence of uninfected CEM cells, the level of IFN-γ in both CD4^+^ and CD8^+^ T cells was negligible (Supplementary Fig. 1).

To determine whether N6/αCD3-αCD28 could stimulate CD8^+^ T cell lysis of HIV infected cells, we examined the lysis of either constitutive or inducible HIV infected T leukemia cell lines: CEM-IIIb and ACH2, respectively^26^. When CD8^+^ T cells from uninfected donors were incubated with either CEM-IIIb or ACH2 cells, they readily lysed the infected target cells in the presence of N6/αCD3-αCD28. As expected, mutations in N6 or CD3 markedly reduced this lysis (Fig. 2C). Incubation with N6/αCD3-αCD28 stimulated higher lysis of CEM-IIIb and ACH2 cells: 2 to 2.4-fold higher compared to a control construct in which the HIV specificity was knocked out and 22- to 14-fold higher compared to a construct in which the CD28 specificity was knocked out, respectively (Fig. 2D). These findings show that the N6 trispecific Ab required engagement of HIV Env and CD3 to stimulate T-cell cytolysis of target cells; and additional engagement of CD28 led to further increased elimination of HIV infected cells. The same mechanism showed effective activation/proliferation of primary human PBMCs in similar settings^33^.

Next, we confirmed activation and antiviral activity of N6/αCD3-αCD28 using lymphocytes from rhesus macaques chronically infected with SHIV_SF162P3_. Autologous CD4^+^ and CD8^+^ T cells from lymph nodes of infected rhesus macaques were co-cultured in the presence of the N6/αCD3-αCD28 or negative controls. Then CD8^+^ T-cell lysis of SHIV_SF162P3_-infected CD4^+^ T cells was examined by quantifying the level of SHIV gag DNA. A significant reduction of SHIV gag DNA was observed in the co-culture with N6/αCD3-αCD28 treatment compared to controls (Fig. 2E; *P* = 0.0001–0.024, Student’s *t*-test).

To determine whether anti-viral activity could be exerted on primary human T cells latently-infected with HIV, we used a model previously shown to mimic latent infection in vitro^35^. Latently-infected, CCL19-treated resting CD4^+^ T cells were incubated with syngeneic CD8^+^ T cells in the presence of the N6/αCD3-αCD28 or controls. Treatment with N6/αCD3-αCD28 significantly reduced expression of intracellular HIV p24 in the CD4^+^ T cells compared to controls. Mutation of CD28 reduced the efficacy of eliminating virus from CD4^+^ T cells compared to the wild type, indicating that CD28 stimulation increased its efficacy. The specificity of this effect was confirmed by comparing infected to uninfected cell. N6/αCD3-αCD28 exerted undetectable levels of uninfected primary T cells cytolysis compared to the negative control, ΔN6/αCD3-ΔαCD28 (Fig. 2F; *P* = 0.0001–0.033, Student’s *t*-test).

### Ex vivo latency activation and lysis of ART-suppressed HIV-1 infected cells

**Fig. 3:**
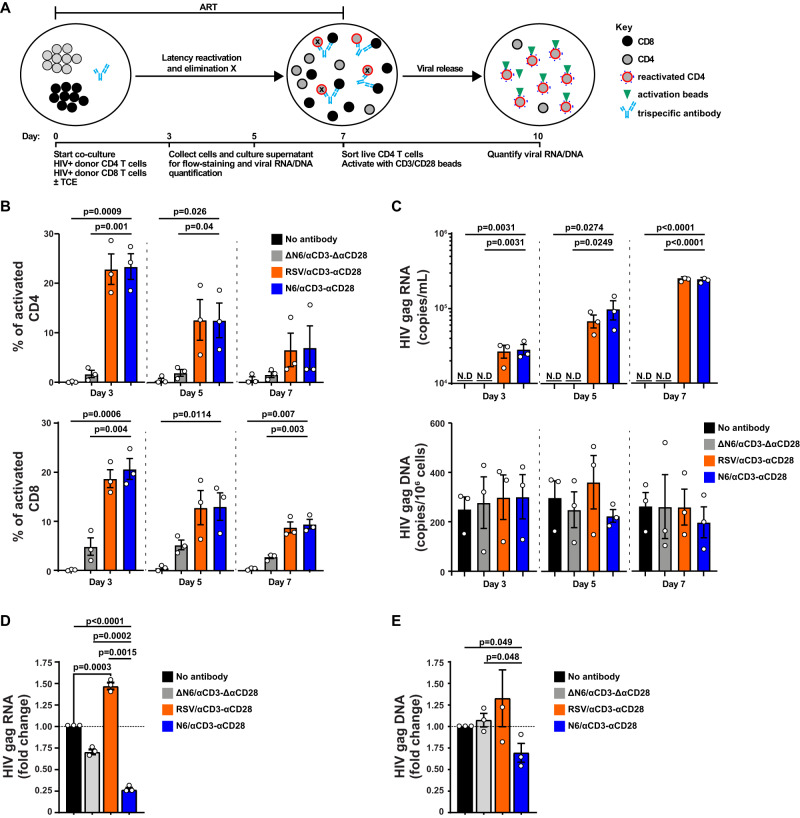
N6/αCD3-αCD28 induces HIV reactivation and redirects T-cell mediated lysis of latently-infected CD4^+^ T cells of ART-suppressed PBMC. **A** Ex vivo latency reversing assay. Sorted autologous CD4^+^ and CD8^+^ T cells from PBMCs of antiretroviral therapy (ART)-treated donors were co-cultured (1:1) and treated with N6/αCD3-αCD28 or control constructs in the presence of ART for 7 days. On day 3, 5, and 7, cells and supernatant from the co-culture were collected and assessed for the induction of T-cell activation, and for the quantification of HIV gag RNA and DNA. On day 7, live CD4^+^ T cells were sorted from the co-culture and stimulated with αCD3/αCD28 activation beads in the absence of both ART and trispecific antibody for 72 h, and the level of HIV gag DNA and RNA were then quantified. **B**, **C** Activation of latently-infected CD4^+^ and CD8^+^ T cells from ART-suppressed HIV infected donors by N6/αCD3-αCD28. Sorted CD4^+^ T cells from ART-suppressed HIV infected PBMC samples were co-cultured with sorted autologous effector CD8^+^ T cells in the presence of trispecific antibodies. Total cells were collected on day 3, 5, and 7 from the co-culture, and were stained with antibodies against CD69 and CD25 for the measurement of activated CD4^+^ and CD8^+^ T cells by flow cytometry (**B**). The culture supernatant was collected on day 3, 5, and 7 for quantification of HIV *gag* RNA and DNA by real time RT-PCR (**C**). **D**, **E** Reduction in viral release from CD4^+^ T cells of ART-suppressed HIV infected donors by N6/αCD3-αCD28. On day 7 of the co-culture, live CD4^+^ T cells were isolated for a viral release assay, in which anti-CD3 and anti-CD28 activation beads were used to stimulate the viral release. The cells and culture supernatant were collected 72 h after for quantification of HIV *gag* RNA (**D**) and DNA (**E**) by real time RT-PCR. The data were plotted as the mean ± SEM. Statistical significance was measured by unpaired, two-tailed Student’s *t*-test with *P* values less than 0.05 considered significant (*n* = 3 biological replicates). N.D not detectable. Source Data are provided as a Source Data file.

In accordance with previous findings using a bispecific antibody^26^, we observed increased expression of HIV Env on the surface of latently-infected CD4^+^ T cells from peripheral blood mononuclear cells (PBMCs) of antiretroviral therapy (ART)-treated donors after incubation with N6/αCD3-αCD28 (Supplementary Fig. 3). To further confirm the activity of N6/αCD3-αCD28 on latently-infected cells from diverse human particpants, we developed an ex vivo latency reversing and killing assay to test whether N6/αCD3-αCD28 could activate and lyse latently-infected T cells. In brief, sorted autologous CD4^+^ and CD8^+^ T cells from ART-suppressed PBMCs (Supplementary Table 1) were co-cultured (1:1) and treated with N6/αCD3-αCD28 or control constructs in the presence of ART for 7 days without the addition of any other cytokines or stimuli (Fig. 3A). On day 3, 5, and 7, cells and supernatant from the co-culture were collected and assessed for the induction of T-cell activation as well as for the quantification of HIV gag RNA and DNA using real-time PCR after normalization for total cell numbers in the sorted cell populations (Fig. 3A). On day 7, live CD4^+^ T cells were sorted from the co-culture and stimulated with αCD3/αCD28 activation beads in the absence of both ART and antibody for 72 h, and the level of HIV gag DNA and RNA were then quantified (Fig. 3A).

During co-culture of CD4^+^ and CD8^+^ T cells in the presence of ART, treatment with either N6/αCD3-αCD28 or RSV/αCD3-αCD28, but not ΔN6/αCD3-ΔαCD28, significantly induced activation of CD4^+^ T cells, as evidenced by increased CD25 and CD69 expression, on day 3 and day 5 when compared to no antibody control (Fig. 3B; *P* = 0.0009–0.026, Student’s *t*-test). The activation level of CD4^+^ T cells was highest on day 3 then decreased over time (Fig. 3B), which coincides with the decline in the proliferation level of CD4^+^ T cells (Supplementary Fig. 4). Similarly, robust induction of CD8^+^ T-cell activation, as evidenced by increased CD25 and CD69 expression, on day 3, 5, and 7 were observed in all antibodies tested when compared to no antibody control (Fig. 3B; N6/αCD3-αCD28 versus No antibody; day 3, *P* = 0.0006; day 5 *P* = 0.0114; day 7, *P* = 0.0007; Student’s *t*-test). N6/αCD3-αCD28 treatment also resulted in the induction of CD8^+^ T-cell effector function, as evidenced by increased CD69 and granzyme B expression, when compared to other antibodies (Supplementary Fig. 4b). Importantly, treatment with either N6/αCD3-αCD28 or RSV/αCD3-αCD28, but not ΔN6/αCD3-ΔαCD28, resulted in detectable and significantly induced levels of HIV gag RNA during the co-culture condition in the presence of ART (Fig. 3C; *P* = 0.0001–0.027, Student’s *t*-test); and the induction of HIV gag RNA was found to increase over time. In contrast, no significant difference in HIV gag DNA levels was observed among the antibodies tested (Fig. 3C). During viral release conditions, in which live CD4^+^ T cells were resorted from the co-culture and restimulated with a strong T-cell activator (αCD3/αCD28 activation beads), both HIV gag RNA and DNA were detectable in all antibodies tested (Fig. 3D, E). However, the level of both HIV gag RNA and DNA was significantly reduced when N6/αCD3-αCD28 treatment was used during the co-culture condition compared to all other antibodies tested (Fig. 3D, E; *P* = 0.0001–0.049, Student’s *t*-test); whereas the level of HIV gag RNA was significantly induced when RSV/αCD3-αCD28 treatment was used during the co-culture condition (Fig. 3D, E; RSV/αCD3-αCD28 versus No antibody; *P* = 0.0003, Student’s *t*-test). Together, these data indicate that the antibody-engagement of both CD3 and CD28 is required to efficiently reactivate latently-infected CD4^+^ T cells, while the engagement of CD3 is the minimal requirement for induction of CD8^+^ T cell effector function. However, only treatment with N6/αCD3-αCD28, which offers engagement of all targets: (1) HIV-1 Env (2) CD3, and (3) CD28, can reactivate HIV gene expression in latently-infected cells and target them for elimination by redirected CD8^+^ T cells; whereas the treatment with RSV/αCD3-αCD28 can only reactivate but not eliminate the latently-infected primary human T cells.

### In vivo safety and pharmacokinetics study with trispecific N6/αCD3-αCD28

**Fig. 4:**
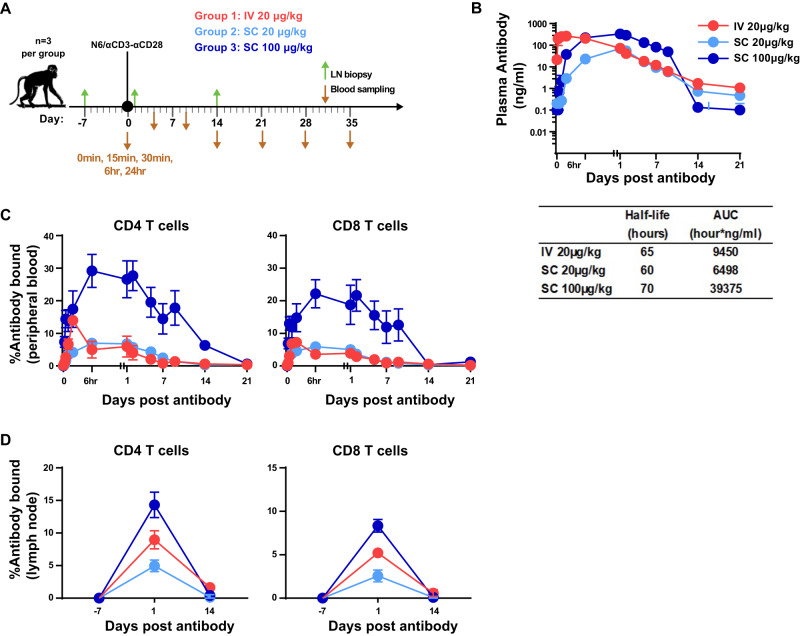
Study design and comparison of the effects of intravenous versus subcutaneous administration of N6/αCD3-αCD28 on pharmacokinetics in naïve rhesus macaques. **A** Rhesus macaques (*n* = 3 per group) received a single dose of N6/αCD3-αCD28 either via intravenous or subcutaneous administration ranging at 20−100 ug kg^−1^ N6/αCD3-αCD28 delivered via either a single intravenous or subcutaneous injection at the dose ranging 20–100 ug kg^−1^. Lymph node and blood samples were collected at various timepoints before, during, and after the administration of N6/αCD3-αCD28. **B** Plasma levels and half-life of N6/αCD3-αCD28 were measured, as described in the “Materials and Method”. **C**, **D** Percentage trispecific N6/αCD3-αCD28 bound to CD4^+^ and CD8^+^ T cells in peripheral (**C**) and in lymph node (**D**). Trispecific N6/αCD3-αCD28 binding to T cells was determined using anti-human IgG4 (Southern Biotech) to measure the percentage of cells with bound N6/αCD3-αCD28 on the indicated subpopulations of T cells by flow cytometry. The data were plotted as the mean of three animals ±SEM. Source Data are provided as a Source Data file.

We performed a safety and pharmacokinetic study to assess the effects of administration of trispecific N6/αCD3-αCD28 in naive rhesus macaques. In a single dose study, naive rhesus macaques were administered N6/αCD3-αCD28 at different concentrations (20 µg kg^−1^ or 100 µg kg^−1^) and routes of administration (intravenous or subcutaneous), then blood and lymph node samples were collected pre, during, and post trispecific antibody infusion to assess the impact on the immune cell activation in both peripheral and secondary lymphoid tissues (Fig. 4A).

We observed that both intravenous and subcutaneous infusions at all dosages tested were well tolerated, as none of the animals experienced clinically evident adverse events. As expected, subcutaneous infusion at 100 µg kg^−1^ resulted in a higher plasma trispecific antibody concentration with similar antibody half-life when compared to either intravenous or subcutaneous infusion at 20 µg kg^−1^ (Fig. 4B). Irrespective of dosages tested, the peak plasma concentration was found at 1 h and 24 h post intravenous and subcutaneous infusions, respectively (Fig. 4B). Both routes were associated with transient decreases in circulating T and B lymphocyte, natural killer and monocyte cell counts that returned to baseline by day 7 and day 35 for the lower dose of 20 µg kg^−1^ and higher dose of 100 µg kg^−1^, respectively (Supplementary Fig. 5). The transient nature of these changes suggested that redistribution rather than depletion of immune cells mediated this effect (Supplementary Fig. 5). We also found that the trispecific N6/αCD3-αCD28 was retained on both CD4^+^ and CD8^+^ T cells in the circulation for at least 48 h when administered at 20 µg kg^−1^ via both routes, and at least 7 days via the subcutaneous route at 100 µg kg^−1^ (Fig. 4C). Importantly, moderate levels of N6/αCD3-αCD28 bound to both CD4^+^ and CD8^+^ T cells in the lymph nodes were also detected 24 h post infusion in all dosages and delivery routes tested (Fig. 4C). In conclusion, these data reveal different pharmacokinetics and dynamics of T-cell interaction between the administration routes, in which the intravenous route offers a more rapid engagement of N6/αCD3-αCD28 with both peripheral CD4^+^ and CD8^+^ T cells whereas subcutaneous route provides a delayed but more sustained effect.

### In vivo impact of trispecific N6/αCD3-αCD28 on immune activation

**Fig. 5:**
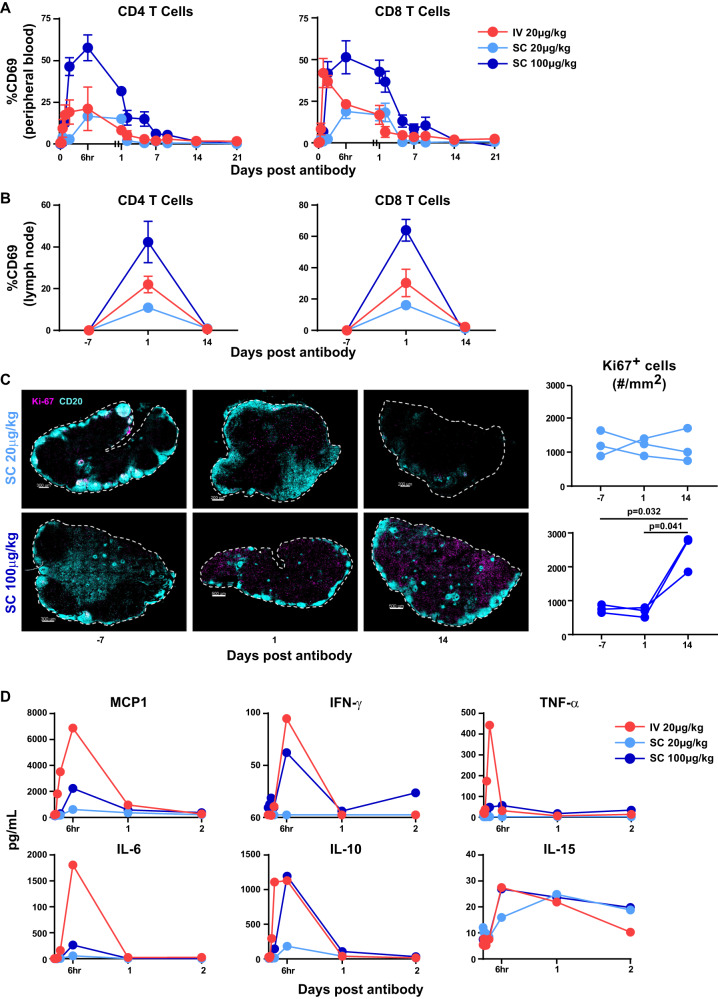
In vivo effects of N6/αCD3-αCD28 on immune activation in naïve rhesus macaques. **A**, **B** T cell activation after N6/αCD3-αCD28 administration. The levels of CD69 expression of CD4^+^ and CD8^+^ T cells in PBMCs (**A**) and lymph node (**B**) was quantified using flow cytometry. **C** Immune activation in lymph nodes after N6/αCD3-αCD28 administration using multiplexed confocal imaging. Representative staining of LN sections (scale bar, 500 mm) from animals received N6/αCD3-αCD28 via subcutaneous administration at 20 ug kg^−1^ (top images) and 100 ug kg^−1^(bottom images), showing CD20^+^ (turquoise), and immune activation marker Ki67 (pink) on days-7, 1 and day 14 post antibody. Quantification of total Ki-67^+^ cells normalized to whole lymph node area (mm^2^) is shown. **D** Increase in inflammatory cytokine levels in plasma after N6/αCD3-αCD28 administration. The levels of inflammatory plasma cytokines were assessed at various time points before and after N6/αCD3-αCD28 administration by a multiplex bead-based assay. The data were plotted as the mean of three animals ±SEM. Statistical significance in (**C**) was measured by ordinary one-way ANOVA, Hom-Sidak’s multiple comparison test with *P* values less than 0.05 considered significant (*n* = 3 biological replicates). Source Data are provided as a Source Data file.

To confirm whether the trispecific N6/αCD3-αCD28 could stimulate the appropriate immune activation in vivo, we first investigated the effect of N6/αCD3-αCD28 on T-cell activation in both peripheral and secondary lymphoid tissues in these animals. As expected, highest induction of both peripheral CD4^+^ and CD8^+^ T-cell activation, as evident by increased CD69 expression, was observed in animals that were administered with 100 µg kg^−1^ via subcutaneous route when compared to lower dose at 20 µg kg^−1^ via either route (Fig. 5A). The peaks of induction occurred at 1 h and 6 h post intravenous and subcutaneous infusion, respectively (Fig. 5A). Similarly, we found that subcutaneous infusion at 100 µg kg^−1^ resulted in the highest induction of both CD4^+^ and CD8^+^ T-cell activation in secondary lymphoid tissue when compared to administration at the lower dose of 20 µg kg^−1^ via either route (Fig. 5B). By combining confocal imaging with quantitative tissue spatial analysis (Supplementary Fig. 6), we characterized how the N6/αCD3-αCD28 administered subcutaneously stimulated cell proliferation/late immune activation via Ki-67 activation in lymph node tissue. Total Ki-67 at day 14 increased in a dose and timely manner with the greatest induction of Ki-67 activation seen at day 14 in the 100 µg kg^−1^ dose group (Fig. 5C; day-7 vs day 14; day 1 vs day 14, *P* = 0.0016, Hom-Sidak multiple comparison test). When comparing the Ki-67 activated cells in follicular and non-follicular areas, Ki-67 activation was significantly induced at day 14 as well in the 100 µg kg^−1^ dose group only. We observed that Ki67-activated CD8^+^ T cells were significantly induced in the follicular area at day 14 (Supplementary Fig. 7, *P* = 0.0252, Hom-Sidak multiple comparison test) in the animals administered the 100 µg kg^−1^ dose compared to those that received 20 µg kg^−1^ dose (Supplementary Fig. 7; *P* = 0.045, Hom-Sidak multiple comparison test).

Stimulation of immune cells by N6/αCD3-αCD28 was further assessed by measuring MCP-1, IFN-γ, TNF-α, IL-6, IL-10, and and IL-15 release in the plasma post infusion. When comparing different routes and concentrations, intravenous infusion of N6/αCD3-αCD28 at 20 µg kg^−1^ induced the highest level of MCP-1, IFN-γ, TNF-α, and IL-6, while subcutaneous infusion at the same concentration resulted in the lowest level for these molecules (Fig. 5d). Subcutaneous infusion at 100 µg kg^−1^ led to moderate induction of MCP-1, IFN-γ, TNF-α, and IL-6 (Fig. 5d). Comparable levels of IL-10 were observed with intravenous infusion at 20 µg kg^−1^ and subcutaneous infusion at 100 µg kg^−1^ (Fig. 5d). Interestingly, similar patterns of induction were observed for most cytokines/chemokines, in which the induction was transient and peaked at 6 h post infusion, except for TNF-α which peaked at 1 h; the levels then returned to pre-treatment levels by 24 h (Fig. 5d). Importantly, the levels of IL-15, which is also known as a marker of immune cell redistribution, were induced at similar magnitudes and patterns by all routes and concentrations tested, in which the peak of induction occurred at 6 h post infusion and the levels remained moderately high up to 7 days post infusion (Fig. 5d). Collectively, these data suggest that subcutaneous infusion of N6/αCD3-αCD28 at 100 µg kg^−1^, when compared to either intravenous or subcutaneous infusion at 20 µg kg^−1^, results in a delayed but sustained interaction with CD4^+^ and CD8^+^ T cells that further provide robust immune cell activation and cytokine/chemokine release in both peripheral and secondary lymphoid tissues.

## Discussion

In this study, we demonstrated that the trispecific N6/αCD3-αCD28 could enhance both reactivation and elimination of latently-infected cells. To our knowledge, this is the first report showing the generation and characterization of a trispecific antibody platform as an alternative HIV-1 therapeutic strategy.

The results from the knock-out control antibodies in these in vitro and ex vivo models confirm that the addition of CD28 specificity contributes to more robust activation of both latently HIV/SHIV infected CD4^+^ as well as CD8^+^ T cells that importantly facilitates effective target recognition and elimination. We hypothesize that following N6/αCD3-αCD28 treatment ex vivo, the latently-infected CD4^+^ T cell reservoir was activated by the anti-CD3/anti-CD28 part of the trispecific antibody, resulting in expression of HIV Env. The HIV Env^+^ cells can then be recognized by the anti-HIV part of the trispecific antibody, which could then mediate the elimination of these infected cells by the activated cytotoxic T cells pulled to the promixicity by the other arms on the trispecific antibody. This mechanism engages T cells that are antigen independent, which may increase the magnitude of anti-HIV activity and therapeutic efficacy. It also demonstrated the feasibility of using a single molecule to both activate and eliminate HIV-1 reservoir cells in an effort to develop a functional cure for HIV/AIDS.

The safety and pharmacokinetics of N6/αCD3-αCD28 was addressed in naïve rhesus macaques and revealed that a single dose of N6/αCD3-αCD28 (dosing 20–100 µg kg^−1^) was safe for use to target and augment robust activation of CD4^+^ and effector CD8^+^ T cells in both peripheral and secondary lymphoid tissues. In accordance with our previous findings with a similar trispecific antibody, αCD38/αCD3-αCD28, which is currently being tested in a clinical trial (NCT04401020)^33^, administration of the trispecific antibody with the inclusion of a monovalent anti-CD28, avoided the potential cytokine release syndrome associated with administering bi-valent anti-CD28 mAb used in previous human studies^36^, reducing non-specific plasma cytokine release and its associated adverse effects. Importantly, high levels of trispecific N6/αCD3-αCD28 bound to CD4^+^ and CD8^+^ T cells was detected in both peripheral and secondary lymphoid tissues, which further resulted in potent CD4^+^ and effector CD8^+^ T cell activation. We detected a high level of T cell activation especially in the follicular areas of the lymph nodes, where HIV reservoirs reside, even after 14 days post infusion when N6/αCD3-αCD28 was given subcutaneously. The detected plasma level of N6/αCD3-αCD28 was also considered sufficient for redirected killing activity against Env-expressing target cells. The plasma level of N6/αCD3-αCD28 started to decline after 2 weeks post infusion, which coincided with the emergence of antidrug antibodies (ADA) response against N6/αCD3-αCD28 (Supplementary Fig. 8). Nevertheless, the results from the single dose regimen supports and suggests that the route of administration can modulate the extent of immune activation, and the subcutaneous route may be a safer and a preferred way to deliver the trispecific N6/αCD3-αCD28 in vivo for activating and targeting T cells and HIV reservoirs.

As previously mentioned, several LRAs have shown the ability to reactivate latency in vivo, however, none of which has demonstrated the capability to eliminate these reactivated cells^37–42^. Therefore, recent shock and kill strategies have proposed and tested the use of LRAs with other one or more “kill” molecules, such as the dual-affinity re-targeting (DART) proteins^43^, or broadly neutralizing antibodies (bnAb)^44^. Despite difference in SHIV model and reservoir size in these studies, the recuring issue with ADA response against the “kill” molecules appeared to be the major hurdle limiting the efficacy of these approaches. Moreover, on-ART viral reactivation was not readily detectable in these studies which rendered it difficult to evaluate whether the antiviral activity observed (or not observed) was due to the “kill” molecules. While beyond the scope of this paper, we suggest that preclinical studies shown to reduce ADA against human antibodies (like B cell depletion^45^) be used when evaluating the therapeutic effect of human antibody-based strategies in SHIV-infected animals.

In summary, our data provide a proof-of-concept demonstrating that trispecific antibody N6/αCD3-αCD28 can safely and successfully be used as a single agent to reactivate and eliminate long-term ART suppressed latently-infected cells ex vivo. We anticipate that, after further preclinical testing in appropriate animal models, future studies assessing trispecific N6/αCD3-αCD28 or similar trispecific antibodies in ART suppressed HIV^+^ individuals, where the development of ADA will not be an issue, would allow for a better assessment of their safety, including potential non-specific activation of T cells, and anti-viral efficacy.

## Methods

### Human participants

Anonymized human PBMCs from normal, healthy donors were obtained through the NIH Clinical Center Department of Transfusion Medicine apheresis program by automated leukapheresis. Signed informed consent from the donors was obtained in accordance with the Declaration of Helsinki and the study was approved by the National Institute of Allergy and Infectious Diseases (NIAID) Institutional Review Board. The donors were compensated as per the study protocol of the NIH Clinical Center Department of Transfusion Medicine apheresis program. Human PBMCs were also obtained from HIV-1-infected donors that were on ART (Supplementary Table 1). Signed informed consent was obtained in accordance with the Declaration of Helsinki and the study was approved by the National Institute of Allergy and Infectious Diseases (NIAID) Institutional Review Board. The donors were compensated as per the study protocol VRC 200 (NIH 03-I-0263) of the Vaccine Research Center (VRC).

### Design of trispecific antibodies

The format for the trispecific antibody was previously described^33^. Briefly, the design of the trispecific antibodies were generated by integrating CODV-Ig bispecific antibody with a conventional antibody arm by heterodimerization using knob-in-hole mutations in the CH3 domain of IgG4 Fc region as described previously^33^. An inactive human IgG4 Fc region was made using mutations that eliminate binding to Fcγ receptors which reduce the release of cytokines associated with non-specific inflammation (IgG4_FALA).

### Construction of expression plasmids

Individual trispecific antibodies were designed based on 3 parameters: (1) Selection of antibody binding sites; (2) Consideration of the position of each binding site; (3) Choice of linkers for the bispecific binding arm. After assembly of the amino acid sequences for each trispecific molecule, four genes for each trispecific antibodies were synthesized using human preferred codons (Cambridge Bio, Brookline, MA, USA), and cloned into a eukaryotic expression vector, as previously described.

### Production and purification of trispecific antibodies

Trispecific antibodies were produced by transient transfection of 4 expression plasmids into Expi293 cells (Thermo Fisher Scientific, Cat. no. A14527) using ExpiFectamine™ 293 Transfection Kit (Thermo Fisher Scientific) according to manufacturer’s protocol, as previously described(ref). Briefly, 25% (w/w) of each plasmid was diluted into Opti-MEM, mixed with pre-diluted ExpiFectamine reagent for 20–30 min at room temperature (RT), and added into Expi293 cells (2.5 × 10^6^ cells/ml). An optimization of transfection to determine the best ratio of plasmids was often used in order to produce the trispecific antibody with good yield and purity. 4–5 days post transfection, the supernatant from transfected cells was collected and filtered through 0.45 µm filter unit (Nalgene). The trispecific antibody in the supernatant was purified using a 3-step procedure. First, protein A affinity purification was used, and the bound Ab was eluted using IgG elution buffer (Thermo Fisher Scientific). Second, the eluted product was dialyzed against PBS (pH7.4) overnight with 2 changes of PBS buffer. Any precipitate was cleared by filtration through a 0.45 µm filter unit (Nalgene) before next step. Third, SEC purification (Hiload 16/600 Superdex 200 pg, or Hiload 26/600 Superdex 200 pg, GE Healthcare) was used to remove aggregates and different species in the preparation. The fractions were analyzed on reduced and non-reduced SDS-PAGE to identify the fractions that contained the monomeric trispecific antibody before combining them. The purified antibody was then aliquoted and kept at −80 °C for long term storage.

### Validation of trispecific antibody binding activities

The binding properties of the purified trispecific antibodies to soluble antigen were analyzed using ELISA. For ELISA assay, the RSC3 was used for assessing N6 binding. Recombinant human CD3γε-IgG1 and CD28-IgG1 proteins were purchased from Cambridge Bio (Cat. No: 03-01-0050, 03-01-0302 respectively. Brookline, MA, USA). The antigens for each binding site in the trispecific antibody were used to coat a 96-well Immuno Plate (Thermo Fisher Scientific) overnight at 4 °C using 2 μg/ml each antigen in PBS (pH7.4). The coated plate was blocked using 5% skim milk+2% BSA in PBS for one hour at RT, followed by washing with PBS + 0.25% Tween 20 three times (Aqua Max 400, Molecular Devices). Serial dilution of antibodies (trispecific and control Abs) were prepared and added onto the ELISA plates (100 μl/well in duplicate), incubated at RT for one hour, followed by washing 5 times with PBS + 0.25% Tween 20. After washing, the HRP conjugated secondary anti-human Fab (1:5000, Cat. No. 109-035-097, Jackson ImmunoResearch Inc) was added to each well and incubated at RT for 30 min. After washing 5 times with PBS + 0.25% Tween 20, 100 μl of TMB Microwell Peroxidase Substrate (KPL, Gaithersburg, MD, USA) was added to each well. The reaction was terminated by adding 50 μl 1 M H2SO4, and OD450 was measured using SpectraMax M5 (Molecular Devices) and analyzed using SoftMax Pro6.3 software (Molecular Devices). The final data was transferred to GraphPad Prism software (GraphPad Software, CA, USA), and plotted as shown. EC50 was calculated using the same software. The binding of the trispecific antibodies to T-cells and HIV Env on the cell surface was performed using human T-cells, rhesus T-cells and chronically HIV-infected (CEM-IIIb) cells. The cells were incubated with the trispecific antibodies (20 mg ml^−1^) for 20 min, and bound proteins were detected with flow cytometry using a fluorescein isothiocyanate-conjugated anti-human IgG (Cat. No. 709-096-149, Jackson Immunoresearch).

### Neutralization assays

Single-round-of-replication Env pseudoviruses were prepared, titers were determined, and the pseudoviruses were used to infect TZM-bl target cells (NIH HIV Reference Reagent Program, Cat. no. ARP-8129) as described previously^34^. Neutralization of monoclonal antibodies was determined using replication-competent SHIV_SF162P3_ and a panel of 6 HIV Env-pseudoviruses. Each NAb was assayed at 5-fold dilutions starting at 50 ug/ml. The neutralization titers were calculated as a reduction in luminescence units compared with control wells and reported as either 50% or 80% inhibitory concentration (IC50 or IC80) in micrograms per milliliter.

### in vitro human PBMC activation

Anonymized human PBMCs from normal, healthy donors were obtained through the NIH Clinical Center Department of Transfusion Medicine apheresis program by automated leukapheresis. Signed informed consent from the donors was obtained in accordance with the Declaration of Helsinki and the study was approved by the National Institute of Allergy and Infectious Diseases (NIAID) Institutional Review Board. These PBMCs were co-cultured for 12 h with either uninfected or HIV-infected (CEM-IIIb) cells in the presence of increasing concentrations of trispecific antibody (0.01−5 nM) and brefeldin A. The cells were then stained for surface expression of T-cell markers (CD3, CD4 and CD8) and activation markers (CD25 and CD69) followed by intracellular staining for cytokines (IFN-gamma, and TNF-alpha) using fluorescently conjugated antibodies (see Supplementary Table 2 for details on the antibodies used). The number of CD4^+^ and CD8^+^ T cells expressing each cytokine or activation marker was determined by running the samples on an LSRFortessaTM X-50 flow cytometer (BD Biosciences) and analyzed with the Flowjo software (Treestar).

### Measurement of in vitro and ex vivo cytolytic activity of trispecific antibodies

CEM-NKR-CCR5 cells (Cat. no. ARP-4376) and latently infected ACH2 cell line (Cat. no. ARP-349) were obtained from the NIH HIV Reagent Program. The chronically HIV-1 infected CEM-NKR-CCR5 cells (CEM-IIIb) and ACH2 cells were labelled with the membrane dye PKH-26 (Cat. No. PKH26GL-1KT, Sigma) and used as target cells in a cytotoxicity assay. These labelled target cells were co-cultured for 12 h at an E:T ratio of 10:1 with enriched human T cells as effector cells in the presence of increasing amounts of the immunomodulatory proteins. The extent of cell lysis in the target cells was determined by staining with a live/dead and apoptotic cell marker, Annexin V (Cat. No. A35110, Life TechnologiesThermo Fisher). Subsequently, the number of dead cells in the labelled target cell population was measured by running the samples on an LSRFortessaTM X-50 flow cytometer (BD Biosciences) followed by analysis using the Flowjo software (Treestar). ex vivo cytolytic activity of trispecific N6/CD28xCD3 was also performed. Briefly, sorted autologous CD4^+^ and CD8^+^ T cells from lymph nodes of chronically SHIV-infected rhesus macaques were used as targets and effectors, respectively, in an ex vivo cytotoxicity assay. They were co-cultured for 12 h at an E:T ratio of 2:1, in the presence of the trispecific antibody (2 nM). Following 12 h co-culture, sorted live CD4^+^ T cells were lysed using the Proteinase K treatment at 55 °C for 1 h followed by inactivation at 95 °C for 5 min. The lysate was spun at high speed to remove debris and 5 ul of lysate was used in a 25-ml PCR reaction. The copy number of SIV gag integrated into the host genome was determined using a quantitative PCR assay. We used the host gene mamu albumin (present in two copies/host genome) to quantify the number of cells in the PCR reaction and calculated the gag copies/cell content accordingly. Gag and mamu albumin were amplified from each sample in parallel reactions (SsoAdvanced Universal Probes Supermix, Bio-Rad). The amplification primers for both the gag (SIV1552-F: GTCTGCGTCATCTGGTGCATTC, SIV1653-R: CACTAGCT GTCTCTGCACTATGTGTTTTG) and mamu albumin (Mamu albumin-F: CCAT GCAGGTGACAGAGACTCT, Mamu albumin-R: TCTCCCCGACAAAGGCATAG) were used at final concentrations of 15 uM and the probes (Gag: 6 fam-CTTCCTCAGTGTGTTTCACTTT CTCTTCTGCG-BHQ1 and Mamu albumin: Vic-TGACACACTGCTG CATGAACCCCA-TAMRA) were at 2.5 uM. Cycling conditions on the CFX96TM Real-Time System (Bio-Rad, Hercules, CA) were as follows: 94 °C for 5 min followed by 45 cycles of 94 °C for 15 s, 60 °C for 60 s. Ramp rates were all 20 °C/s. Tenfold dilutions of DNA extracted from 3D8 cells and BLCL cells were used as a standard curve for gag and mamu albumin, respectively. the CFX ManagerTM software version 3.1 was used to determine copy number for each sample.

### in vitro latency model

Anonymized human PBMCs from normal, healthy donors were obtained through the NIH Clinical Center Department of Transfusion Medicine apheresis program by automated leukapheresis. Signed informed consent from the donors was obtained in accordance with the Declaration of Helsinki and the study was approved by the National Institute of Allergy and Infectious Diseases (NIAID) Institutional Review Board. Resting CD4^+^ [CD4^+^CD25^−^CD69^−^] and CD8^+^ T cells were sorted from the PBMC samples. The CD8^+^ T cells were kept in culture in IL-2 (10 IU/ml) containing media. The resting CD4^+^ T cells were first cultured in the presence of CCL19 (100 nM) for 3 days. These CD4^+^ T cells were then infected with HIV BaL (multiplicity of infection = 0.1) by spinoculation with centrifugation of the cells with the virus at 1,200 g for 2 h at room temperature. These CD4^+^ T cells were then washed twice with media and cultured for 3 days in the presence of IL-2 (10 IU/ml). The infected resting CD4^+^ T cells were then co-cultured with allogeneic CD8^+^ T cells in the presence of the trispecific antibody (2 nM) for 12 h. The co-cultures were then stained with fluorescently conjugated antibodies against T-cell markers (CD3, CD4 and CD8), activation markers (CD25 and CD69), and p24 gag (KC57, Coulter) proteins (see Supplementary Table 2 for details on the antibodies used), followed by flow cytometric analysis using the Flowjo software (BD Biosciences).

### ex vivo latency reversing and killing assay

Human PBMCs were obtained from HIV-1-infected donors that were on ART (Supplementary Table 1). Signed informed consent was obtained in accordance with the Declaration of Helsinki and the study was approved by the National Institute of Allergy and Infectious Diseases (NIAID) Institutional Review Board. Sorted autologous CD4^+^ and CD8^+^ T cells from these PBMC were co-cultured at an E:T ratio of 1:1, in IL-2 (10 IU/ml) and efavirenz (40 nM) containing media with the presence of the trispecific antibody (5 nM). Cells and culture media were collected on day 3, 5, and 7 for quantification of HIV gag DNA and RNA, respectively. On day 7, CD4^+^ T cells were resorted from the co-culture for viral release assay. In brief, sorted CD4^+^ T cells were stimulated with anti-CD3 and anti-CD28 activation beads (Miltenyi Biotec) in the absence of the trispecific antibody. After 3 days of stimulation, cells and culture media were collected for quantification of HIV gag DNA and RNA, respectively. The co-cultures were then stained with fluorescently conjugated antibodies against T-cell markers (CD3, CD4 and CD8), activation markers (CD25, CD69, granzyme B), and proliferation marker (Ki67) (see Supplementary Table 2 for details on the antibodies used), followed by flow cytometric analysis using the Flowjo software (BD Biosciences).

### Safety and pharmacokinetics (PK) study in naïve rhesus macaques

The trispecific N6/aCD3-aCD28 was delivered via either a single intravenous or subcutaneous injection at the dose ranging 20–100 µg/kg, in naïve male and female rhesus macaques aged 2–4 years (*n* = 3 at each dose). Due to insufficient statistical power, sex was not considered in the study design and analysis. Lymph node and blood samples were collected at various timepoints before, during, and after the administration of N6/aCD3-aCD28. All animal experiments under the animal study protocol, VRC-17-721, were reviewed and approved by the Animal Care and Use Committee of the Vaccine Research Center, NIAID, NIH, and all animals were housed and cared for in accordance with the local, state, federal and institute policies in an American Association for Accreditation of Laboratory Animal Care-accredited facility at the NIH.

### Measurement of trispecific antibody concentration in NHP plasma

Measurements were done using Simoa® homebrew assay (Quanterix) with HD−1 device. Biotinylated human CD2819−152 - murine IgG2a Fc fusion protein (Ancell, 508-030) was used for detection. For capture, bead-conjugated mouse monoclonal [HP6025] antibody to human IgG4 Fc (Abcam, ab99820) was used to measure IgG4 FALA N6/αCD28(5.11A1)/αCD3(20G6) antibody concentration; or bead-conjugated human recombinant RSC3 protein (in-house produced) was used to measure simianized IgG4 FALA N6/αCD28(5.11A1)/αCD3(20G6) antibody concentration. Plasma samples were typically diluted 25-fold in Sample Diluent (Quanterix). Trispecific antibodies diluted in Sample Diluent containing the corresponding fraction (e.g., 4%) of naïve monkey plasma were used as calibrators to create a calibration curve.

### Measurement of immune cell levels, T-cell activation, and trispecific antibody-bound to T cells during treatment

Whole-blood samples were obtained from animals at various times during administration of N6/aCD3-aCD28. The whole-blood was directly stained with fluorescently conjugated antibodies against CD3, CD4, CD8, CD20, CD159a, CD14, CD16, HLA-DR, and CD69 (see Supplementary Table 2 for details on the antibodies used) to assess the distribution of immune cells and T-cell activation. After incubation with the antibodies, the blood was then lysed with RBC lysing buffer (BD Biosciences) and run on an LSRFortessaTM X-50 flow cytometer (BD Biosciences) to assess the proportions of peripheral immune cells at different times during treatment. Trispecific N6/aCD3-aCD28 binding to T cells was determined by staining PBMC and lymph node cells with anti-human IgG4 (SouthernBiotech) to measure the percentage of cells with bound N6/aCD3-aCD28 on CD4^+^ and CD8^+^ T cells by flow cytometric analysis using the Flowjo software (BD Biosciences).

### Immune activation in lymph nodes using multiplexed confocal imaging and quantitative image analysis

Lymph nodes were prepared for imaging studies by removing any excess fat after biopsy, then fixed in 4% PFA (Electron Microscopy Science) -PBS 1X overnight at room temperature (RT). After embedding in paraffin blocks, 5 μm thickness sections were mounted on SuperFrost slides (Thermo Fisher Scientific). For detection of immune activation markers with multiplexed confocal imaging, slides were first baked at 60 °C for 1 h followed by deparaffinization baths of xylene (twice, 2 min each) then rehydrated in decreasing concentration ethanol baths (100%, 95%, 80%, 0%, 2 min each). Antigen retrieval was completed in Borg Decloaker RTU (Biocare Medical) in a pressure cooker at 110 °C for 15 min. Slides were permeabilized and blocked for 1 h with blocking solution (PBS 1X, 0.3% Triton X-100, 1% BSA). Primary antibody against CD3 (clone F7.2.38, Cat. No. M7254, Dako), was incubated overnight at 4 °C. Slides were washed (PBS 1X three times, 15 min each) before incubation with secondary antibody Goat anti-mouse IgG1a (AlexaFluor 594, Cat. No. A21125, Thermo Fisher Scientific) for 2 h at RT. After another wash step, slides were blocked with 10% normal goat, mouse sera for 1 h at RT. Conjugated antibodies against CD20 (clone L26 ef615-conjugated, Cat. No. 42-0202-82, Thermo fisher), Ki67 (clone B56 AF700-conjugated, Cat. No. 561277, BD Bioscience), CD4 (polyclonal goat, AF488-conjugated, Cat. No. FAB8165G, R&D) were incubated for 2 h at RT followed by an additional wash step. Slides were then counterstained for nuclei with JOPRO (Cat. No. J11373, Invitrogen) for 20 min at RT then mounted with Fluoromount-G (Cat. No. 0100-01, Southern Biotech) and allowed to cure at 33 °C. Images were acquired using a Nikon C2 confocal microscope (40X objective, 1.40 NA) and processed with Imaris 9.5.0 (Bitplane). Histo-cytometry was conducted on confocal images to quantify various cell populations^46^. Briefly, individual cells were segmented using Imaris Surface Creation tool. The average voxel intensity of each channel, position x, position y, sphericity, and volume for each surface was imported into Microsoft Excel to generate a common-separated value file for FlowJo. Once imported into FlowJo, specific cell populations are gated. Total single cell population was defined by sphericity vs volume followed by defining the tissue boundaries with position x vs position y. Gate for Ki-67 was defined within the tissue boundaries with mean intensity of the marker vs sphericity. Follicular areas were defined based on CD20^+^ to define individual follicles. Boolean “and” gate was used to combine the follicle gates to define the follicular area and the “or” gate was used to generate the non-follicular area. Gates of populations of interest were dragged into the follicular and non-follicular areas. For gating consistency, a negative staining (full panel minus one marker of interest) was generated for Ki-67. Data were normalized by area (mm^2^) to account for difference in tissue size. Statistical analyses were conducted in Prism software v.8. For in vivo effects of trispecific N6/αCD3-αCD28 on immune activation, one-way ANOVA Holm-Sidak’s multiple comparisons test was used to compare between multiple timepoints within a treatment group. Two-way ANOVA followed by Sidak’s multiple comparison was used to compare timepoints between two treatment groups.

### Quantitation of cytokines and chemokines in the plasma

Plasma cytokines were measured using the Milliplex Non-human Primate Cytokine/Chemokine kit (Millipore) using the Luminex xMAP multiplexed bead system (Millipore), according to the manufacturer’s instructions. Results obtained from the Luminex xMAP system were analyzed automatically by the Luminex xPONENT software program (Millipore) using a standard curve derived from recombinant cytokine and chemokine standards.

### Measurement of anti-drug antibody responses

Anti-drug antibody (ADA) responses were evaluated as follows. Plasma from macaques that had been administered the trispecific bNAb were diluted with PBS containing 5% skim milk, 2% BSA and 0.05% Tween 20. Five-fold serial dilutions ranging from 1:50 to 1:781250 of these plasmas were then added in duplicate wells to 96-well ELISA plates coated with 2 µg/ml of the trispecific N6/aCD3-aCD28. The plate was incubated for 1 h at room temperature followed by a PBS-T (PBS with 0.05% Tween-20) wash. Bound monkey IgGs were then probed with a horseradish peroxidase (HRP)-conjugated anti-monkey IgG, Fc-specific (Southern Biotech) for 30 min at room temperature. The plate was then washed and SureBlue TMB (Kirkegaard & Perry Laboratories, Gaithersburg, MD) substrate was added. Once color was developed (typically 15 to 20 min), stopping buffer (1 N H2SO4) was added and the optical density at 450 nm was read. Endpoint titer was calculated by determining the lowest dilution that had optical density greater than five-fold of that in the background wells.

## Supplementary information

Supplementary Information

## Data Availability

The data sets generated during and/or analyzed during the current study are available and provided within the Article, Supplementary information, or Source Data file. Source data are provided with this paper.
