# Supplementary material for: Trispecific antibody targeting HIV-1 and T cells activates and eliminates latently-infected cells in HIV/SHIV infections
Source: Nat Commun. 2023 Jun 22;14:3719. doi: 10.1038/s41467-023-39265-z (PMC10287722; doi:10.1038/s41467-023-39265-z)
Supplement: Supplementary file 1 — Supplementary Information [file 41467_2023_39265_MOESM1_ESM.pdf]

Supplementary information:

Full Title: Trispecific antibody targeting HIV-1 and T cells activates and eliminates latently-infected cells in HIV/SHIV infections

Wanwisa Promsote[1], Ling Xu[2,3], Jason Hataye[1], Giulia Fabozzi[1], Kylie March[1], Cassandra G. Almasri[1], Megan E. DeMouth[1], Sarah E. Lovelace[1], Chloe Adrienna Talana[1], Nicole A. Doria-Rose[1], Krisha McKee[1], Sabrina Helmold Hait[1], Joseph P. Casazza[1], David Ambrozak[1], Jochen Beninga[2], Ercole Rao[2], Norbert Furtmann[2], Joerg Birkenfeld[2‡], Elizabeth McCarthy[1], John-Paul Todd[1], Constantinos Petrovas[1], Mark Connors[4], Andrew T. Hebert[2], Jeremy Beck[2], Junqing Shen[2], Bailin Zhang[2], Mikhail Levit[2], Ronnie Wei[2,3], Zhi-yong Yang[2,3], Amarendra Pegu[1], John R. Mascola[1], Gary J. Nabel[2,3], and Richard A. Koup[1]

Affiliations:

[1]Vaccine Research Center, National Institute of Allergy and Infectious Diseases, National Institutes of Health, Bethesda, MD, USA.

[2]Sanofi, 640 Memorial Dr., Cambridge MA, 02139, USA

[3]ModeX Therapeutics Inc., 22 Strathmore Road, Natick, MA 01760, USA

[4]NIAID, NIH, Bethesda, MD, 20892, USA

[‡]Current address: Perspix Biotech GmbH, FiZ Frankfurt Innovation Center Biotechnology, Altenhoeferallee 3, 60438 Frankfurt, Germany.

*Correspondence to: rkoup@nih.gov, gary.nabel@modextx.com

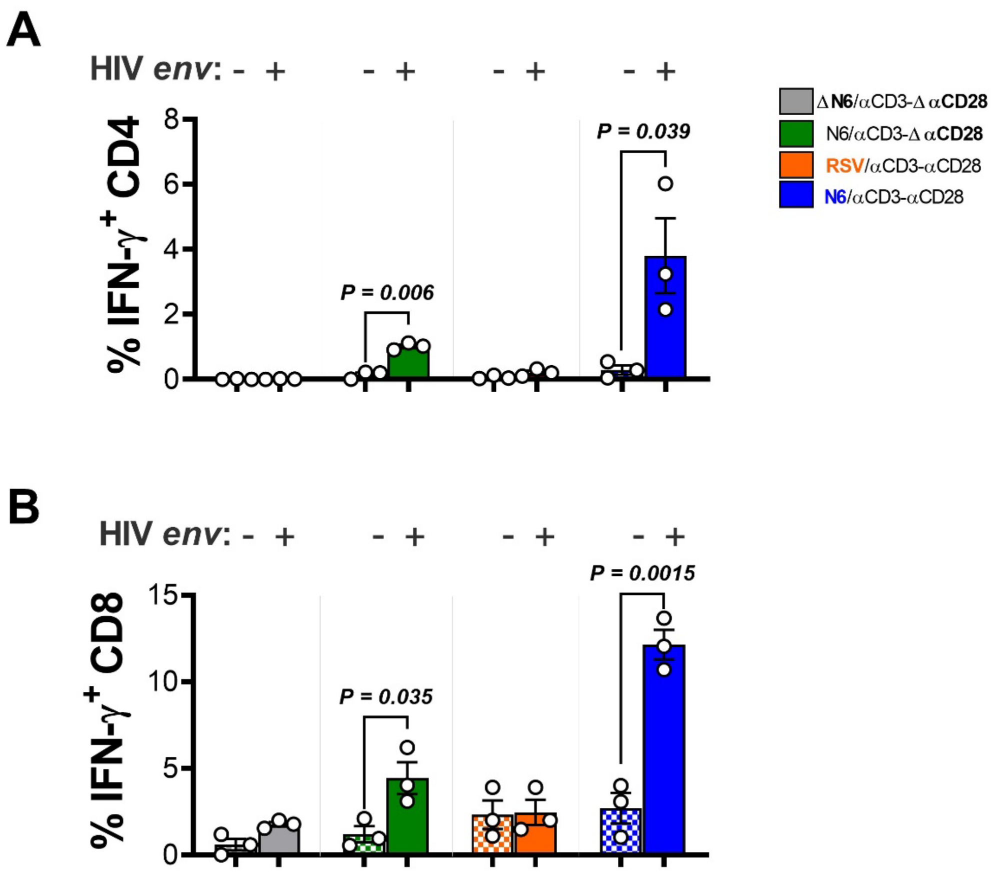

Supplementary Figure 1. N6/αCD3-αCD28 enhances the HIV-specific T cell activation. Expression of intracellular IFN-γ by $CD4^+$ and $CD8^+$ T cells activated by N6/αCD3-αCD28. Enriched human T cells were co-cultured with either uninfected (HIV *env*-) or HIV-infected CEM (HIV *env*+) cells in the presence of 2nM of the indicated T cell engagers and Brefeldin A for 16 hours. The T cells were then stained with antibodies against IFN-γ and the percentage of T cells expressing IFN-γ was measured using flow cytometry. The data were plotted as the mean ± SEM. Statistical significance was measured by unpaired, two-tailed Student's t-test with P values less than 0.05 considered significant (n = 3 biological replicates). Source Data are provided as a Source Data file.

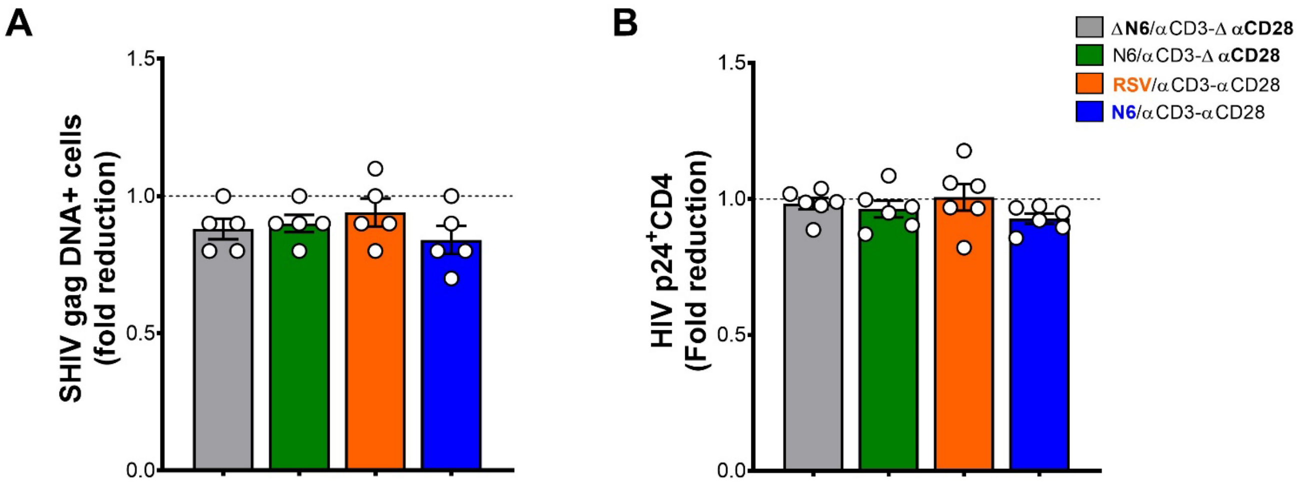

Supplementary Figure 2. The effects of trispecific antibodies on T cell mediated lysis of SHIV- and HIV-1 infected cells in the absence of CD8$^+$ T cells. (A) Levels of SHIV *gag* DNA copies in CD4$^+$ T cells from SHIV-infected LN when treated with N6/αCD3-αCD28 and indicated controls. Sorted CD4$^+$ T cells were cultured in the presence of trispecific antibodies for 12 hours. CD4$^+$ T cells were then resorted from the culture for quantification of SHIV g*ag* DNA. **(B)** Number of latently-infected primary CD4$^+$ T cells when treated with N6/αCD3-αCD28 and indicated controls. Resting CD4$^+$ T cells (CD4$^+$CD25$^-$CD69$^-$) were sorted from human PBMCs and infected with HIV BaL after culture in the presence of CCL19 for 3 days. These CD4$^+$ T cells were then treated with indicated trispecifc antibodies for 14 hours. The expression of intracellular p24 of CD4$^+$ T cells was then measured by flow cytometry. The data were plotted as the mean ± SEM. Statistical significance was measured by unpaired, two-tailed Student's t-test with P values less than 0.05 considered significant (n = 5 and 6 biological replicates for **(A)** and **(B)**, respectively). Source Data are provided as a Source Data file.

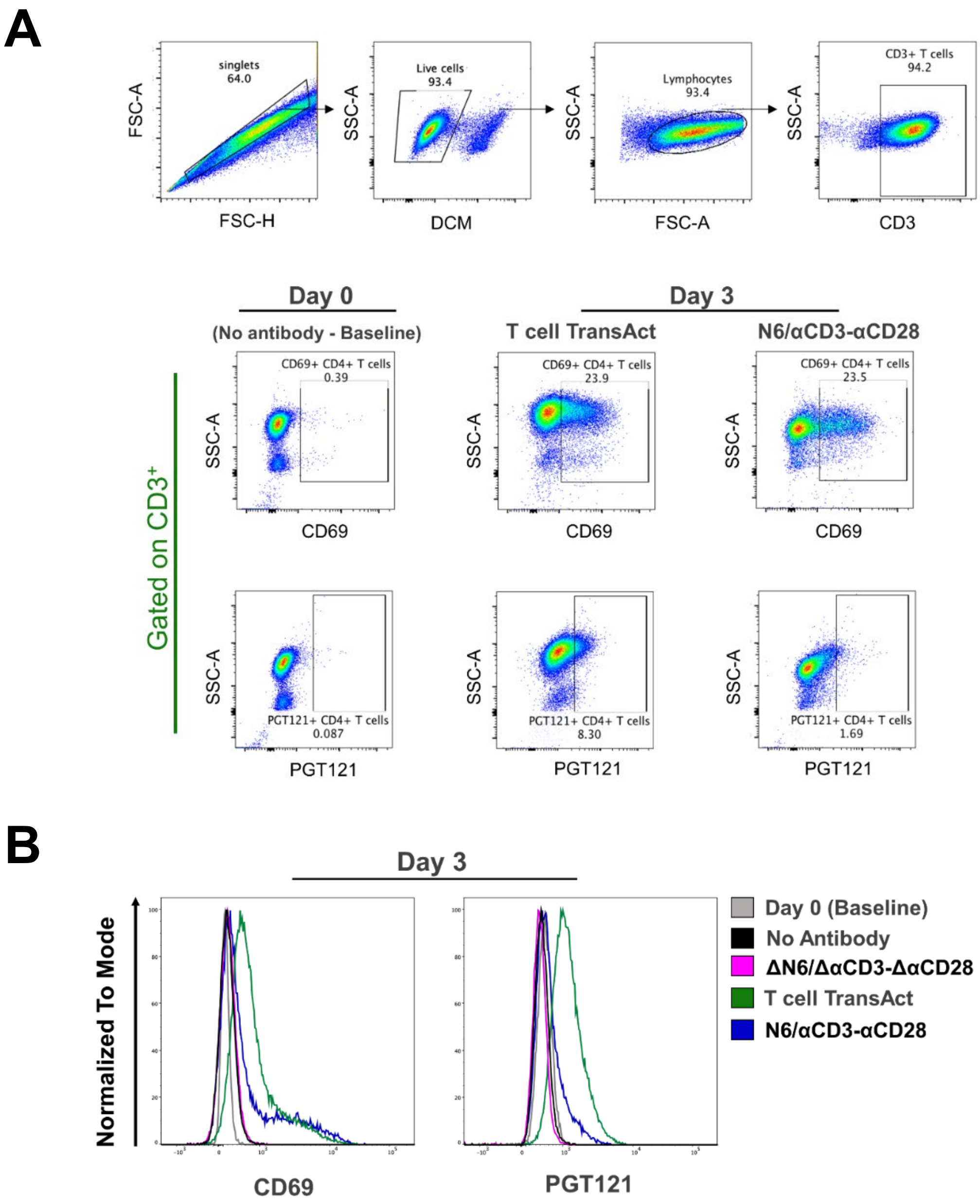

Supplementary Figure 3. N6/αCD3-αCD28 induces activation and HIV Env expression of latently-infected CD4$^+$ T cells of ART-suppressed PBMC. Sorted CD4$^+$ T cells from peripheral blood mononuclear cells (PBMCs) of antiretroviral therapy (ART)-treated donors were treated with 5nM of N6/αCD3-αCD28 or ΔN6/ΔαCD3-ΔαCD28, and T cell TransAct (positive control, Miltenyi) in the presence of indinavir for 3 days without the addition of any other cytokines or stimuli. Cells were then collected on day 3 and assessed for the activation and HIV Env expression of latently-infected CD4$^+$ T cells by flow cytometry. The CD4$^+$ T cells were stained with antibodies against viability marker (DCM), CD3, and CD69 (activation marker). The expression of HIV Env on the surface of CD4+ T cells was measured by flow staining with a fluorescently labelled PGT121 antibody. **(A)** Gating strategy for flow cytometry analysis to evaluate activation and HIV Env expression of latently-infected CD4$^+$ T cells of ART-suppressed PBMC. **(B)** Representative data from one donor showing increased T cell activation and HIV Env expression of CD4+ T cells following treatment with N6/αCD3-αCD28 and controls.

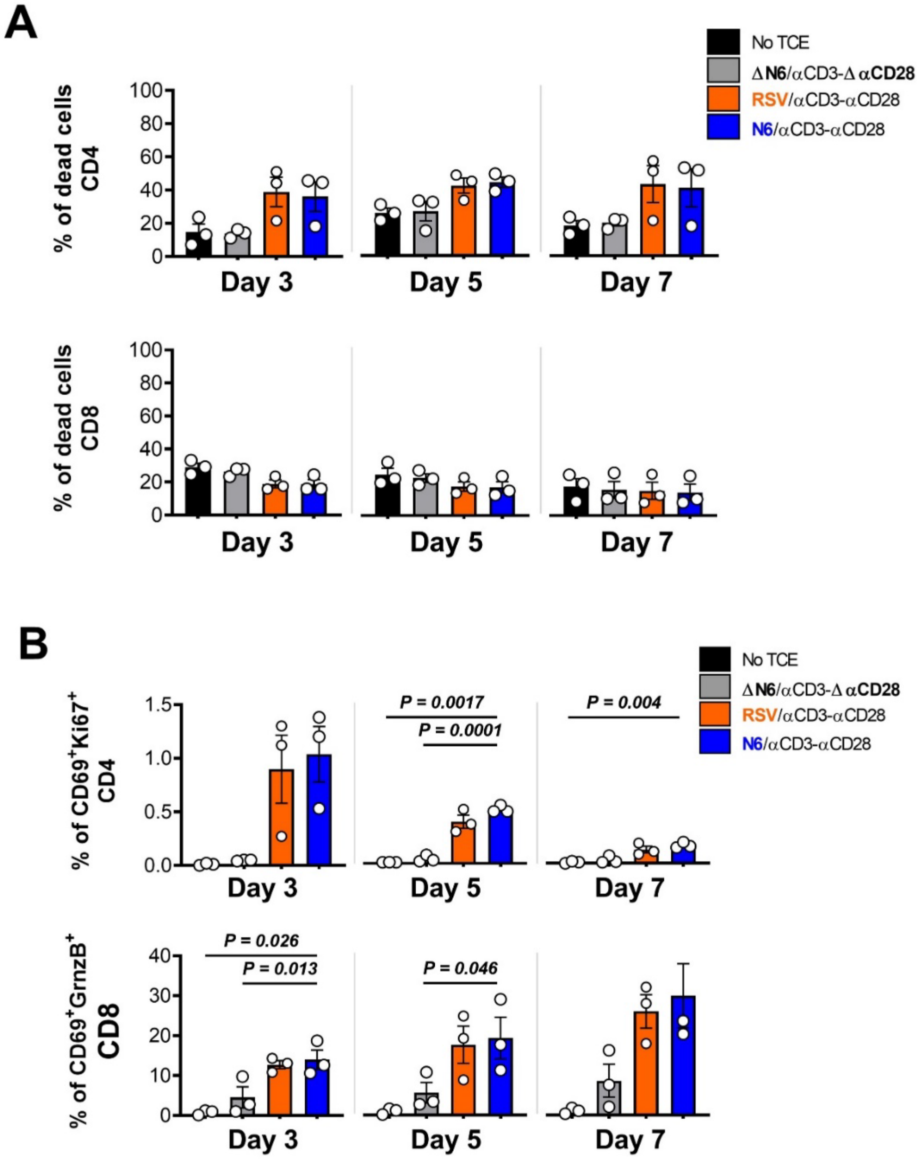

Supplementary Figure 4. N6/αCD3-αCD28 induces HIV reactivation of latently-infected CD4$^+$ T cells and enhances effector CD8$^+$ T cells of ART-suppressed PBMC cells. *Ex vivo* latency reversing assay was performed in which sorted autologous CD4$^+$ and CD8$^+$ T cells from peripheral blood mononuclear cells (PBMCs) of antiretroviral therapy (ART)-treated donors were co-cultured (1:1) and treated with N6/αCD3-αCD28 or control constructs in the presence of ART for 7 days without the addition of any other cytokines or stimuli. On day 3, 5, and 7, cells from the co-culture were collected and assessed for the activation and proliferation of latently-infected CD4$^+$ and CD8$^+$ T cells by flowcytometry. The CD4$^+$ and CD8$^+$ T cells were stained with antibodies against CD69, Ki67, granzyme B and viability marker, and the percentage of dead cells as measured by a live/dead cell marker **(A)** and activated proliferating **(B)** CD4$^+$ and CD8$^+$ T cells were measured using flow cytometry**.** The data were plotted as the mean ± SEM. Statistical significance was measured by unpaired, two-tailed Student's t-test with P values less than 0.05 considered significant (n = 3 biological replicates). Source Data are provided as a Source Data file.

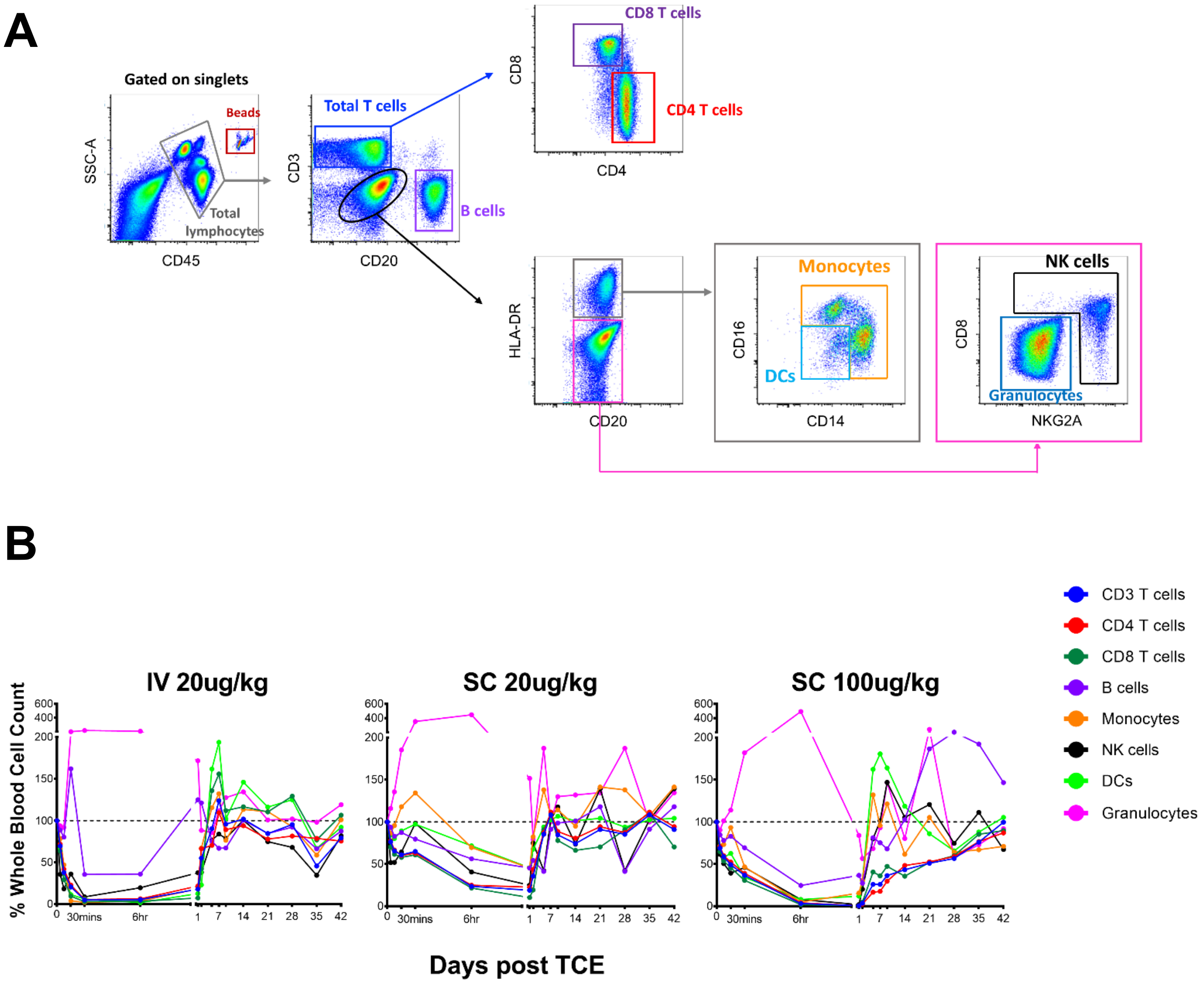

Supplementary Figure 5. ***In vivo*** **administration of N6/αCD3-αCD28 results in redistribution of periphery immune cells of naive rhesus macaques. (A)** Gating strategy for flow cytometry analysis of whole blood staining to evaluate immune cell redistribution in periphery. **(B)** Flow cytometry immunophenotyping analysis showing transient distribution of circulating immune cell subsets following administration of N6/αCD3-αCD28 via intravenous or subcutaneous at the dose of 20 μg kg^{-1} or 100 μg kg^{-1}. The data were plotted as the mean percentage (n = 3 animals/group). Source Data are provided as a Source Data file.

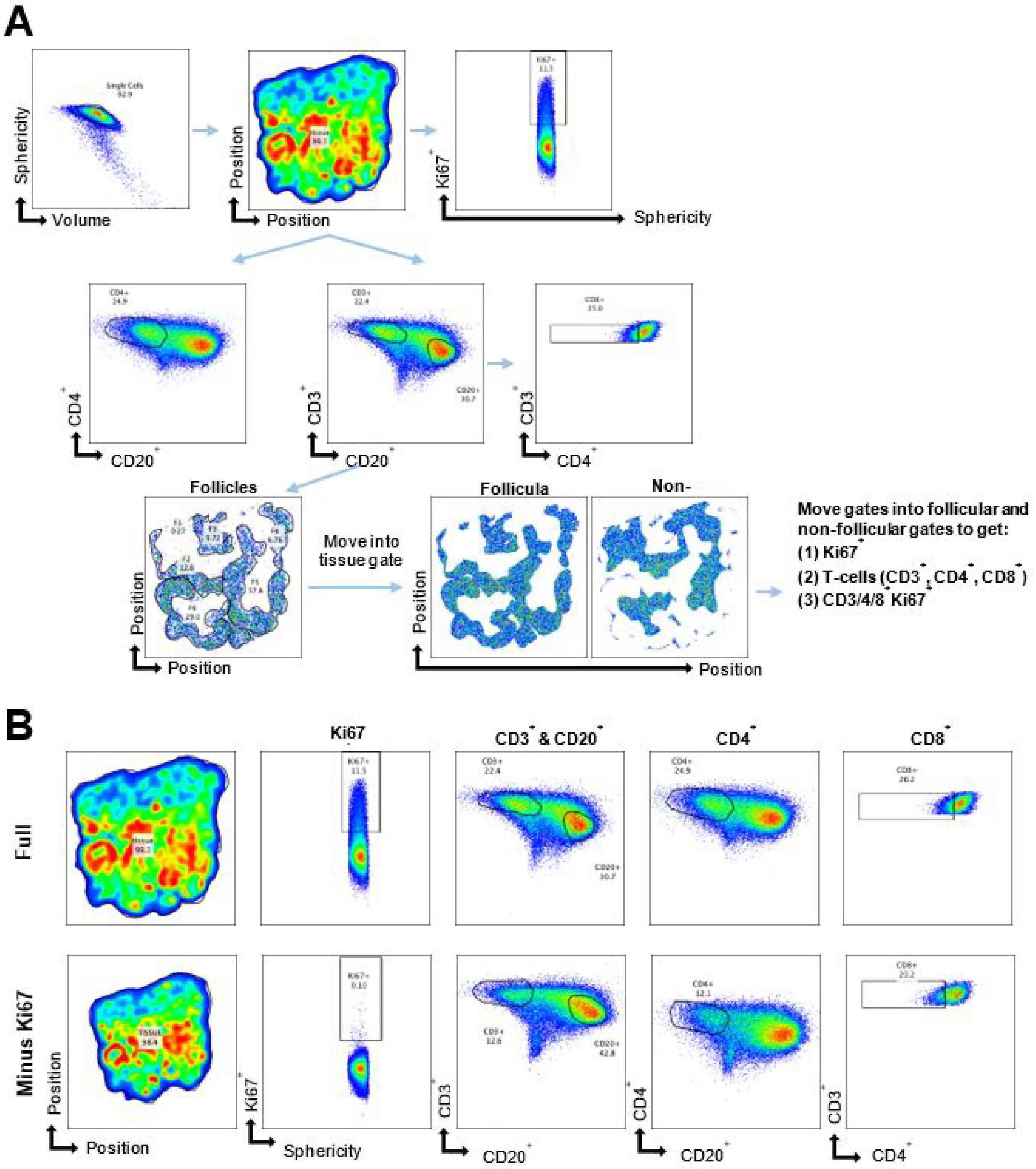

Supplementary Figure 6. Gating strategy for quantitative tissue spatial analysis to characterize how the N6/αCD3-αCD28 administered subcutaneously stimulated cell proliferation/late immune activation via Ki67 activation in lymph node tissue.

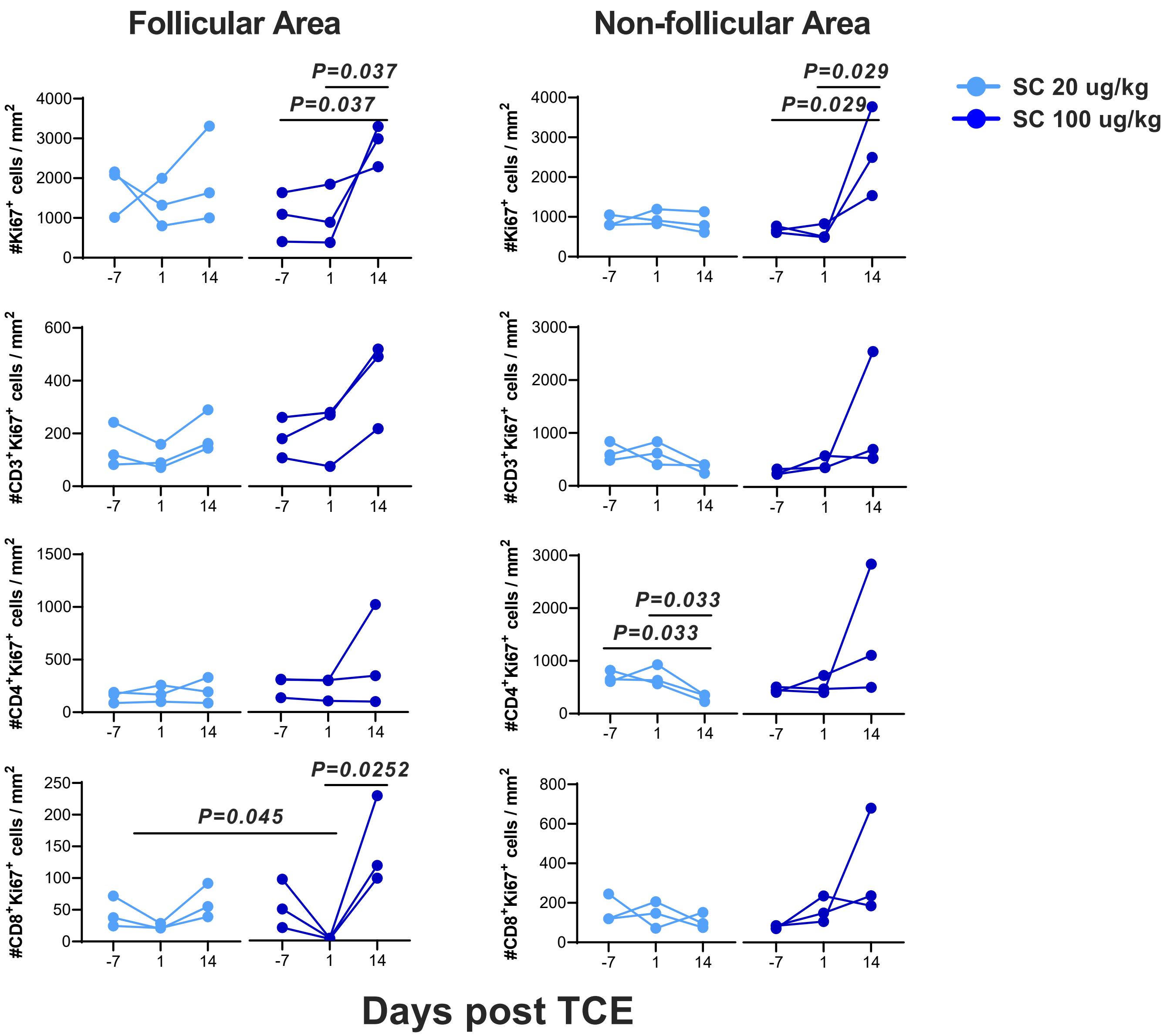

Supplementary Figure 7. Immune activation in lymph nodes of naïve rhesus macaques after subcutaneous administration N6/αCD3-αCD28 using multiplexed confocal imaging. Quantification of Ki67-activated $CD3^+$ T cells, $CD4^+$ T cells and $CD8^+$ T cells normalized to whole lymph node area (mm^2) from animals received N6/αCD3-αCD28 via subcutaneous administration at 20ug kg^{-1} and 100ug kg^{-1} on days-7, 1 and day 14 post antibody are shown. Ki67-activated $CD8^+$ T cells were significantly induced in the follicular area at day 14 in the animals administered the 100 µg kg^{-1} dose compared to those that received 20 µg kg^{-1} dose. For *in vivo* effects of trispecific N6/αCD3-αCD28 on immune activation, one-way ANOVA Holm-Sidak's multiple comparisons test was used to compare between multiple timepoints within a treatment group. Two-way ANOVA followed by Sidak's multiple comparison was used to compare timepoints between two treatment groups. P values less than 0.05 considered significant (n = 3 biological replicates). Source Data are provided as a Source Data file.

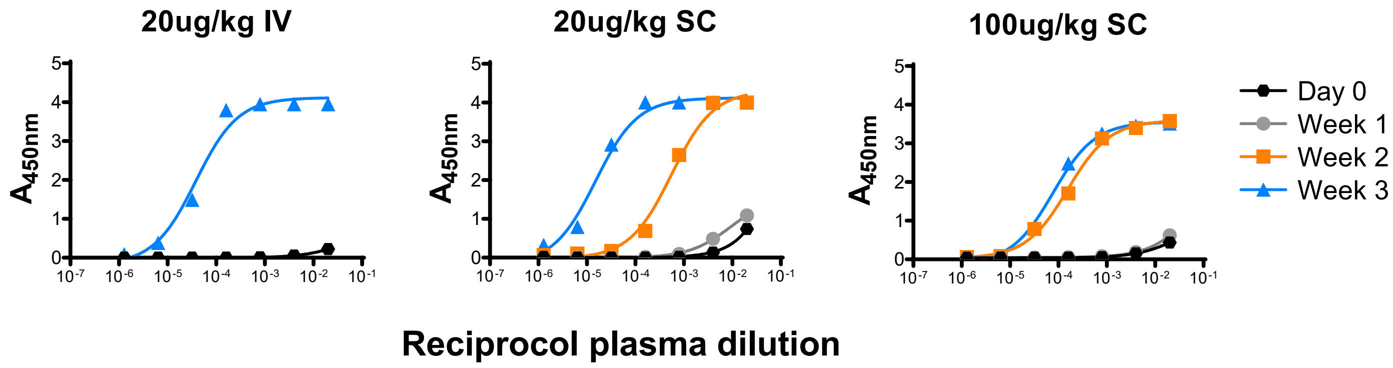

Supplementary Figure 8. Anti trispecific N6/αCD3-αCD28 plasma IgG in naïve macaques. Binding of plasma from indicated time points to N6/αCD3-αCD28 after administration via intravenous or subcutaneous at the dose of 20 μg kg^{-1} or 100 μg kg^{-1} as detected by an ELISA-based assay. Source Data are provided as a Source Data file.

Supplementary Table 1. HIV-1 infected donors on anti-retroviral therapy

Donor	Year of HIV diagnosis	Year started therapy	ARVs	Day of visit	CD4 T cell count/l of blood	Plasma viral load (copies/ml)
1	1991	Unknown	TAF/FTC/ATV/DTG/COBI	7/27/2017	731	122
2	2012	Unknown	TAF/FTC/EVG/COBI	9/6/2017	752	<20
3	1984	Unknown	ABC/3TC/ATV	6/17/2008	352	361

Supplementary Table 2. Antibodies used in flow cytometry

Marker	Fluorochrome	Clone	Supplier	Catalog no.
CD14	BV510	M5E2	Biolegend	301842
CD16	BUV496	3G8	BD Biosciences	564653
CD16	BV510	3G8	Biolegend	302048
CD20	BUV737	2H7	Biolegend	564432
CD25	BV421	M-A251	BD Biosciences	562442
CD28	ECD	CD28.2	Beckman Coulter	6607111
CD3	APC-Cy7	SP34-2	BD Biosciences	557757
CD4	BUV805	SK3	BD Biosciences	564910
CD45	FITC	D058-1283	BD Biosciences	557803
CD69	BV605	FN50	Biolegend	310938
CD8	BV785	RPA-T8	Biolegend	301046
DCM	Zombie UV	-	Biolegend	423108
DCM	Aqua	-	Invitrogen	L34966
Granzyme B	APC	GB12	thermo Fisher	MHGB05
HLA-DR	PECy5.5	TU36	Life Technologies	MHLDR18
IFNγ	FITC	B27	BD Biosciences	554700
Ki67	BV605	Ki67	Biolegend	350522
NKG2A	PE-Vio770	REA110	MACS Miltenyi Biotec	130-105-647
TNF	BV785	Mab11	Biolegend	502948
